# Core–Shell Rubber Nanoparticle-Modified CFRP/Steel Ambient-Cured Adhesive Joints: Curing Kinetics and Mechanical Behavior

**DOI:** 10.3390/ma17030749

**Published:** 2024-02-04

**Authors:** Abass Abayomi Okeola, Jorge E. Hernandez-Limon, Jovan Tatar

**Affiliations:** 1 Department of Civil and Environmental Engineering, University of Delaware, Newark, DE 19716, USA; aokeola@udel.edu (A.A.O.); eljorge@udel.edu (J.E.H.-L.); 2 Department of Civil and Environmental Engineering, Center for Composite Materials, University of Delaware, Newark, DE 19716, USA

**Keywords:** CFRP, steel, adhesive, core–shell rubber, nanoparticles, silane, curing kinetics, bridges, repair, strengthening

## Abstract

Externally bonded wet-layup carbon fiber-reinforced polymer (CFRP) strengthening systems are extensively used in concrete structures but have not found widespread use in deficient steel structures. To address the challenges of the adhesive bonding of wet-layup CFRP to steel substrates, this study investigated the effect of core–shell rubber (CSR) nanoparticles on the curing kinetics, glass transition temperature ($T_{g}$) and mechanical properties of ambient-cured epoxy/CSR blends. The effects of silane coupling agent and CSR on the adhesive bond properties of CFRP/steel joints were also investigated. The results indicate that CSR nanoparticles have a mild catalytic effect on the curing kinetics of epoxy under ambient conditions. The effect of CSR on the $T_{g}$ of epoxy was negligible. Epoxy adhesives modified with 5 to 20%wt. of CSR nanoparticles were characterized with improved ductility over brittle neat epoxy; however, the addition of CSR nanoparticles reduced tensile strength and modulus of the adhesives. An up to 250% increase in the single-lap shear strength of CFRP/steel joints was accomplished in CSR-modified joints over neat epoxy adhesive joints.

## 1. Introduction

Externally bonded carbon fiber-reinforced polymer (CFRP) composites are rapidly gaining acceptance in repairing and retrofitting of deficient steel and concrete structures [1,2]. Aging steel bridges, in particular, can benefit from CFRP strengthening to prolong their service life, especially in the United States where there are about 600,000 bridges, 34% of which are made of steel [3]. Approximately 40% of those bridges require repair to prevent further deterioration [4]. While past studies have demonstrated that precured CFRP laminates can be used to improve the strength and serviceability of steel bridges [5,6,7], precured laminates are rigid and not easily adaptable to irregular configurations that are present on most bridges. On the other hand, the flexibility of wet-layup CFRP allows easy application to different geometrical configurations. The similarity between the adhesive resin used for wet-layup CFRP and that used for bonding CFRP to substrates facilitates the application of the strengthening technique. Wet-layup CFRP has been extensively used to strengthen concrete members [8,9,10]. However, the widespread adoption of this technology in steel bridges faces a hurdle due to the unsuitability of epoxy resins designed for concrete substrates when applied to steel substrates. The primary challenge to the direct adoption of existing commercial wet-layup systems is modifying the composition of the adhesive to impart increased fracture toughness without compromising its mechanical and thermal properties. The following outlines the specific challenges and requirements that must be addressed to enable more widespread use of wet-layup systems in steel structures:

Firstly, epoxy (mostly used for CFRP applications) is cured via an exothermic reaction that may require an external heat supply to ensure the full conversion of the epoxy and the achievement of the desired mechanical, thermal, and chemical properties [2,11]. At relatively low curing temperatures, the degree of crosslinking between the polymer chains of epoxy is low, and the amount of free volume (microscopic space available) in the polymer structure is high [12]. Increased free volume enabled by incomplete cure of ambient-cured epoxy combined with presence of hydrophilic hydroxyl groups in the polymer network makes epoxy susceptible to moisture absorption, which leads to plasticization [2,12]. The moisture absorption further increases free volume and polymer chain mobility, which lowers the crosslinking density and, consequently, the glass transition temperature ($T_{g}$) while also reducing the elastic modulus and strength of epoxy [12]. Although exposure to temperatures above the cure temperature (but below $T_{g}$) will continue to increase the degree of crosslinking [2,11], direct application of heat is impractical due to the size of the structural elements on most bridges, and the sometimes hard-to-reach construction joints. Therefore, ambient-cured cured epoxy adhesives must possess sufficiently high $T_{g}$ to ensure adequate short- and long-term performance [2,13].

Secondly, although the mechanical properties of the existing wet-layup CFRP systems are appropriate for steel bridge retrofitting (i.e., high stiffness), the viscosity of the adhesives used to bond CFRP to the concrete is not suitable for bonding CFRP to steel. The porosity of concrete allows low-viscosity epoxy to penetrate the substrate, permitting epoxy to establish mechanical interlock in addition to chemical bonds to concrete [10,14]. Steel, on the other hand, has no appreciable porosity and, as a result, high-viscosity epoxy is required to achieve adequate bonding, and prevent epoxy dripping during overhead applications. The addition of core–shell rubber nanoparticles to epoxy, explored in this study, is a proven means of increasing the viscosity of the adhesive and should facilitate wet-layup CFRP application [15,16].

Finally, the desired failure mode for CFRP externally bonded to concrete structures is cohesive failure of concrete [17]; to achieve this failure mode, brittle adhesives with high strength and elastic moduli are usually preferred over adhesives with significant inelastic energy capacity. On the contrary, recent evidence suggests that in CFRP/steel bonded joints, the preferred bond failure mode is the cohesive failure of the adhesive where fracture toughness of adhesives is a principal factor governing adhesive bond performance over adhesive’s tensile strength [18,19,20]. Hence, adopting the existing CFRP systems (originally developed for concrete applications) in steel structures may be feasible if suitable modifications can be made to high-modulus/high-strength adhesives traditionally used in concrete structures.

Fracture toughness of an epoxy adhesives can be improved by incorporating rubber-based additives such as a carboxyl-terminated copolymer of butadiene acrylonitrile (CTBN) [21,22,23], amine-terminated butadiene acrylonitrile (ATBN) [24], CSR nanoparticles, and natural liquid rubbers [25] to the epoxy base resin. However, the introduction of rubber-based additives to epoxy matrix can result in a significant reduction of $T_{g}$ [26], adhesive strength and elastic modulus of epoxy [15,24]. CSR nanoparticles consisting of a soft rubbery core (like polymethyl methacrylate or PMMA) surrounded by a hard shell can be functionalized to improve the compatibility between the base resin and nanoparticles [15], eliminating phase-separation challenges observed with CTBN, while improving fracture toughness to epoxy [15,27].

**Figure 1:**
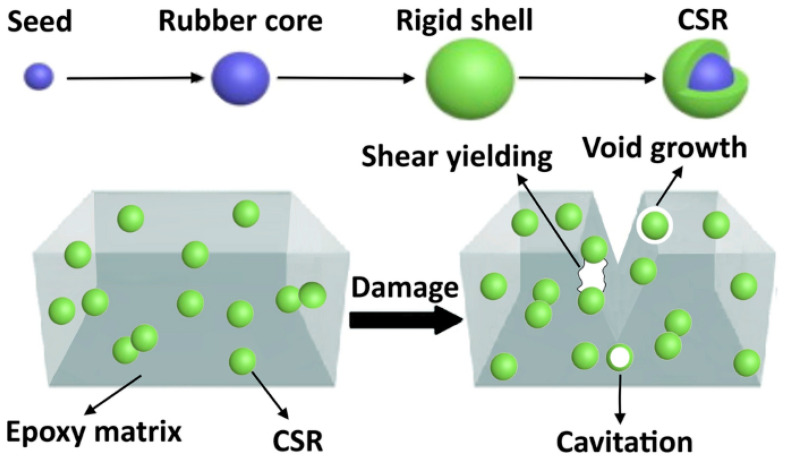
Toughening mechanism of CSR in epoxy matrix. (Reprinted with permission from Springer Nature Customer Service Centre GmbH: Springer, Journal of Materials Science, toughening of epoxy resin systems using core–shell rubber particles: a literature review by Mousavi et al. (2021) [29]).

Toughening mechanisms enabled by CSR nanoparticles in the epoxy matrix include the following: (i) localized cavitation in the rubber or at the rubber/matrix interface, (ii) plastic shear yielding in the matrix, due to the interaction between the rubber and the stress field at the crack tip, (iii) stretching and tearing of embedded rubber particles, and (iv) enlargement of fractured surface area due to the introduction of multiple fracture path [15,21,28]. Figure 1 shows a graphic representation of some of the possible toughening mechanisms resulting from the CSR nanoparticles.

Aside from modifying epoxy with nanoparticles, CFRP/steel adhesive bond performance can also be improved by modifying the surface of steel using mechanical treatments (e.g., grit blasting and sandblasting) or functionalization with coupling agents such as titanate, zirconium compounds, and silanes [21,29,30,31,32]. Silanes are the most commonly used coupling agents for improving the adhesive bond strength of adherends to substrates [31]. Silanes have two functional groups, one that forms covalent bonds with inorganic substrates (steel) and the other one that establishes a covalent bond with an organic functional group in the adhesive [32,33]. Thus, silanes serve as an “indirect bridge”, establishing covalent bonds between adhesives and substrates. Substrate silane treatment can also improve the durability of adhesive bonds [5], silane-induced covalent bonds are stronger than hydrogen bonds (bonds formed in the absence of silane) and are not as susceptible to water disruption [32,34].

The existing studies on CSR-modified adhesive joints considered primarily aerospace industry applications, where manufacturing procedures and curing conditions are significantly different from applications of wet-layup CFRP in infrastructure. No studies have reported on the effect of CSR nanoparticles on the properties of ambient-cured adhesives. Therefore, to evaluate the benefits of blending CSR nanoparticles with ambient-cured epoxy typically used in wet-layup CFRP application combined with the silane surface treatment, this study investigated the following: (1) curing kinetics and $T_{g}$ of epoxy/CSR blends; (2) tensile strength, elastic modulus, and elongation of epoxy/CSR blends; and (3) lap shear strength and failure mode of CFRP/steel adhesive joints.

## 2. Materials and Methods

The methods used to achieve the aims of the study include the following: (1) contact angle measurement to investigate the effect of silane treatment on the surface energy of steel; (2) isothermal calorimetry and differential scanning calorimetry (DSC) to observe the effect of CSR loading ratio (ranging from 0 to 25%wt.) on the isothermal curing kinetics of epoxy/CSR blends within a range of temperatures typical for infrastructure applications (25 °C to 55 °C); (3) mechanical tests to measure the effect of CSR loading ratio on the tensile strength, elastic modulus, and elongation of the epoxy/CSR blends; and (4) single-lap shear tests on CFRP/steel joints to understand the effect of CSR nanoparticles and silane surface treatment on the joint strength and failure mode. The range of loading ratios of CSR nanoparticles was selected to be representative of the typical range of values encountered in the literature, while the test temperatures were chosen as representative of in-service field conditions during the adhesive cure.

### 2.1. Silane Steel Surface Treatment

The effectiveness of silane treatment of steel substrate towards achieving improved adhesive strength was recorded by measuring the surface energy of steel plates treated with silane. Low-carbon steel plates (ASTM A108), measuring approximately 15.2 cm in length and 2.5 × 0.64 cm in cross-sectional dimensions were used (Table 1). The steel surface was first roughened using 180-grit sandpaper for 5 min and cleaned with acetone to remove any contaminant. After allowing the samples to dry, aminopropyltriethoxysilane Dow Xiameter™ OFS-6011 dissolved in 5%wt. isopropyl alcohol was wiped onto roughened steel substrates and allowed to dry under room temperature. The contact angle of Diiodomethane and deionized water on the prepared surfaces were measured using Sigma 7000. Approximately one μL droplets of diiodomethane and distilled water were placed at 10 separate locations on each sample. Surface energy was then computed using Young–Dupre’s and Fowkes’s equation [35,36]. Steel surfaces with and without silane treatment had surface energy of, 63.5 ± 5.6 mN/m and 32.8 ± 3.3 mN/m, respectively. The 93% increase in surface energy of the silane-treated surface implies a hydrophilic surface that will increase the “wettability” of adhesives applied on steel substrates [37].

### 2.2. Isothermal Calorimetry

TAM air isothermal microcalorimeter (TA instruments—Waters L.L.C., New Castle, DE, USA) was used to obtain the heat flow during the curing of neat epoxy (NE) and epoxy/CSR blends. NE (Epon 826, Hexion, Columbus, OH, USA) was cured with amine hardener (Jeffamine D-230, Huntsman, Maple Shade, NJ, USA). Epoxy monomer and hardener were mixed in a 100:33 ratio by weight to achieve the stoichiometric equivalence between the functional groups (100:33 by weight). The mixture was vigorously stirred for 15 min before being poured into glass ampoules for isothermal studies.

Epoxy/CSR blends were prepared by dispersing CSR masterbatch (Kaneka Kane Ace^TM^ MX-960, Kaneka, Pasadena, TX, USA) in Epon 826 to achieve a 5, 10, 15, 20, and 25%wt. loading ratio of CSR nanoparticles in the resin. The masterbatch consists of 300 nm CSR nanoparticles with a polysiloxane core and a polymethyl methacrylate (PMMA) shell dispersed at 25%wt. in a Bisphenol-A-based resin (JER828). According to the manufacturer, the CSR particles were lightly functionalized to ensure low viscosity of the masterbatch and facilitate dispersion. Table 2 shows the properties of epoxy, CSR, amine hardener, and JER 828. The dispersion was performed by heating a mixture of the CSR masterbatch and Epon 826 at 80 °C to reduce the viscosity of the mixture. The hot mixture was then subjected to high shear mixing for 20 min to facilitate dispersion of CSR nanoparticles, as recommended by the manufacturer. The mixture was allowed to cool down to room temperature before the hardener was added and stirred for 15 min. About 5 ± 0.2 g of the samples of NE and epoxy/CSR blend were carefully poured into glass ampoules and cured at 25 °C, 35 °C, 45 °C, and 55 °C. The average heat flow of three samples was collected at a rate of 13.6 kHz and normalized using the weight of the sample (excluding the weight of inert CSR nanoparticles).

### 2.3. Differential Scanning Calorimetry

The effect of CSR loading ratio and isothermal curing temperature on the glass transition ($T_{g}$) of NE and epoxy/CSR blends were evaluated using Discovery DSC calorimeter (TA instruments). Approximately 17 ± 4.5 mg of NE and epoxy/CSR blend were prepared using the same sample preparation approach described in Section 2.2 and poured into aluminum pans. The samples were then gently placed in glass ampoules and cured in TAM Air calorimeter at 25 °C, 35 °C, 45 °C, and 55 °C for 7 days. Differential scanning calorimetry experiments were conducted on the cured samples in a nitrogen atmosphere by first cooling the samples to −30 °C, then heating them up to 270 °C at a rate of 10 °C/min. The heat flow and temperature data were acquired at 300 Hz and $T_{g}$ was reported as the mid-point temperature of the glass transition range in the first heat run.

To determine the reaction enthalpy ($∆H_{0}$) of neat epoxy and CSR masterbatch, freshly prepared (uncured) samples were heated from −60 to 300 °C at a 10 °C/min rate. Reaction enthalpy was then calculated by integrating the exothermic peak area. It was determined that both neat epoxy and CSR masterbatch (corrected for the weight of CSR) have a reaction enthalpy of approximately 440 J/g, consistent with data reported in the literature for similar epoxy systems [38]. The same test was used to determine the $T_{g}$ of uncured systems ($T_{g0}$).

Finally, to construct master plots (i.e., establish a relationship between $T_{g}$ and conversion), neat epoxy and epoxy/CSR blends were first cured under isothermal conditions at 25 °C for 4, 8, 12, and 24 h, then tested in DSC by heating from −60 to 300 °C at a 10 °C/min rate. Residual reaction enthalpy ($∆H_{r}$) was obtained by integrating the area under the exothermic peak. Conversion of each sample (α) was then calculated as follows:

(1) $$\alpha =\frac{∆H_{0}-∆H_{r}}{∆H_{0}}$$

The $T_{g}$ was calculated as the mid-point temperature of the glass transition range in the first heat run. To obtain $T_{g}$ at full cure ($T_{g\infty }$), fresh samples were heated from −60 to 300 °C at 10 °C/min, held at 300 °C for 5 min, then cooled to 0 °C/min, and finally heated to 300 °C at 10 °C/min. $T_{g\infty }$ was computed at the second heat run, ensuring that no exotherm was present (which indicates full cure of the resin).

### 2.4. Epoxy Tensile Test

**Figure 2:**
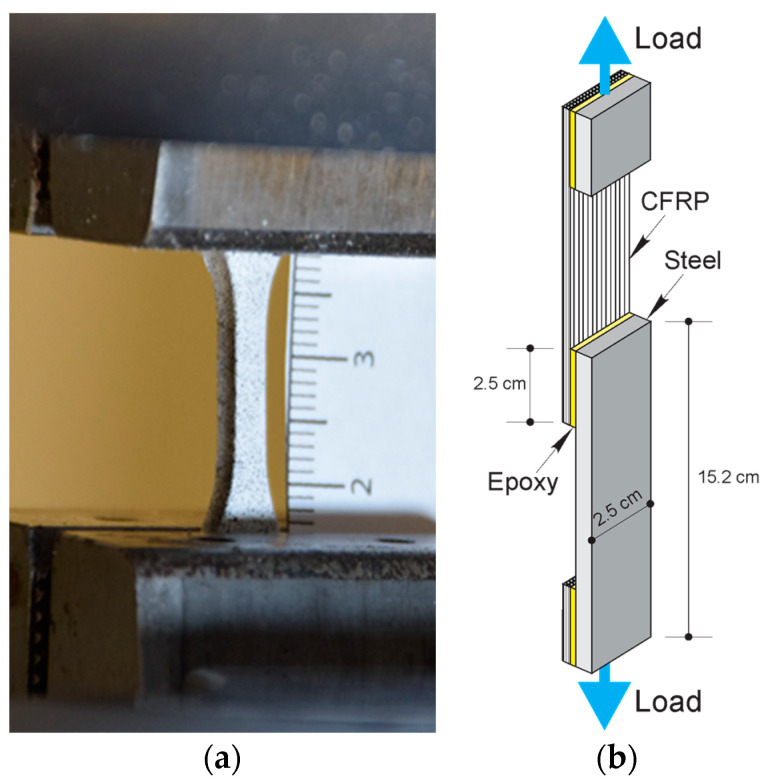
Test specimens: (**a**) gripped Type V dogbone specimen (scale in cm); (**b**) single-lap shear test epoxy adhesives.

Epoxy dogbone specimens (Type V, ASTM D638) were prepared by injecting epoxy adhesives into reusable silicone rubber molds (coated with a Frekote 770-NC release agent) with a 30 mL polypropylene syringe (Figure 2a). The prepared specimens were cured for 7 days under standard laboratory conditions before testing (23 ± 2 °C and RH 50 ± 10%). Tensile tests on epoxy dogbones were performed using an MTS Universal Testing Machine under a constant displacement rate of 1 mm/min until the specimen failed. Load was recorded with a 100 kN MTS load cell and strain was measured using a VIC 2-D digital image correlation (DIC) system. The DIC setup consisted of a Grasshopper^®^ 3 CCD camera with an AF Zoom-Nikkor 70–300 mm telephoto zoom lens having a spatial resolution of 2448 × 2048 pixels. The average length–pixel ratio of the system is approximately 3.45 µm/pixel. The displacement error is 0.01 pixel. The lighting source, placed approximately 1 m away from the test sample, consisted of a Utilitech Pro 2-Light 36-Watt LED stand. DIC images were collected at 15 Hz.

### 2.5. Single-Lap Shear Tests

Single-lap shear test samples were prepared using steel plates and CFRP. CFRP composites were prepared by the hand-layup method from a unidirectional carbon fiber fabric with a fiber weight of 644 g/m^2^. The CFRP laminates were cured for a minimum of 7 days under standard laboratory conditions (23 ± 2 °C and RH 50 ± 10%). The properties of the laminate are summarized in Table 3.

Painter’s tape was applied to the ends of the steel plate prepared in Section 2.1 leaving only the 2.5 × 2.5 cm area exposed for bonding to CFRP. The epoxy adhesive was then applied to the surface of the cured CFRP laminate, and the steel plate was carefully placed over the composite and allowed to cure for 7 days under standard laboratory conditions (23 ± 2 °C and RH 50 ± 10%). Following the initial specimen cure, grip tabs were applied as shown in Figure 2b.

Single-lap shear test specimens (Figure 2b) were separated into two groups: (1) with silane treatment and (2) without silane treatment. The control group utilized neat epoxy (NE+0%), while CSR-modified groups had 5%, 10%, 15%, and 20%wt. of CSR by weight of the epoxy resin. Each group of specimens consisted of 5 replicates. CSR-modified groups were labeled as NE+5%, NE+10%, NE+15%, and NE+20%, according to the %wt. of CSR.

Single-lap shear experiments were performed under 1.3 mm/min displacement control in an MTS Universal Testing Machine until failure. Load and machine crosshead displacement were recorded. Following each test, the actual area of the adhesive joint was measured with a caliper and photographic evidence of failure mode was collected. The average shear strength of each adhesive joint was computed using Equation (2):

(2) $$\tau_{avg}=\frac{P_{max}}{A_{meas}}$$

where $P_{max}$ is the maximum load recorded at specimen failure; and $A_{meas}$ is the actual postmortem measured area of the adhesive joint.

## 3. Statistical Analysis

To assess if there were statistically significant differences among test groups, one-way ANOVA was conducted, followed by post hoc *t*-tests. The initial ANOVA was carried out with a significance level set at 0.05. Subsequently, post hoc t-tests were employed to identify specific pairs of groups displaying statistically significant differences in their average values, with a significance level of 0.05. To mitigate the risk of making a Type I statistical error during these post hoc tests (which can occur when conducting multiple comparisons), Bonferroni correction was implemented. This correction adjusts the significance level for each individual comparison, making it more stringent and helping to maintain an appropriate overall error rate in the analysis.

In Section 4 of the paper, qualitative statements that compare various test groups are made. These statements were informed by the outcomes of the statistical analyses herein described.

## 4. Results and Discussion

### 4.1. Effect of CSR on Curing Kinetics of Epoxy

**Figure 3:**
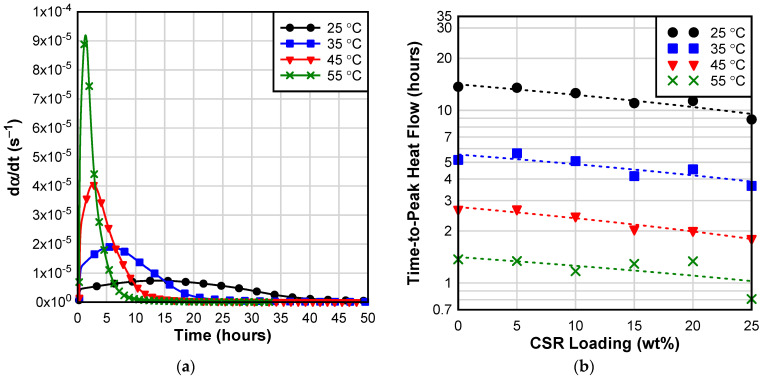
Isothermal calorimetry: (**a**) reaction rate of NE as a function of time and curing temperature; (**b**) effect of CSR loading on time-to-peak heat flow at different curing temperatures [dashed lines are only to guide eyes.].

Isothermal calorimetry was adopted to evaluate the effect of curing temperature and CSR loading on the curing kinetics of epoxy modified with CSR nanocomposites. Figure 3a shows an example of the rate of reaction (computed as the differential of the normalized heat flow) as a function of curing time and temperature. As expected, the reaction rate initially increases rapidly due to the autocatalytic reaction mechanism. Once the peak reaction rate is reached, the reaction decelerates and eventually becomes asymptotic. The reaction rate is influenced by the curing temperature—the peak reaction rate increases with isothermal curing temperature. In addition, the time-to-peak heat flow was observed to decrease with the curing temperature and CSR loading (Figure 3b) suggesting that CSR nanoparticles may have a catalytic effect on epoxy cure. This catalytic effect is likely due to the functional groups present on the CSR surface; unfortunately, the CSR manufacturer reported that the CSR nanoparticles were “lightly functionalized” but the details regarding the specific functional groups present on the surface of the nanoparticles were not disclosed. A study conducted by Pramanik et al. [39] reported that the high surface area of the nanosized CSR particles, coupled with the presence of acid functional groups (e.g., -COOH) can accelerate epoxy–amine reaction, especially at low conversions; a similar catalytic effect was observed by Li et al. [40]

To further interrogate the observed catalytic effect of CSR on the curing reaction (Figure 3b), isothermal curing kinetics were modeled using Kamal equation [41]:

(3) $$\frac{d\alpha }{dt}={\left(k_{1}+k_{2}\alpha^{m}\right)\left(1-\alpha \right)}^{n}$$

where α is conversion; t is time; $k_{1}$ and $k_{2}$ are non-catalytic and autocatalytic rate constants, respectively; and m and n are constants, where m+n is also known as the reaction order. Kamal equation generally provides an acceptable fit in the initial stages of the reaction. However, as the reaction progresses, the mobility of polymeric chains becomes restricted, so the reaction ultimately becomes diffusion controlled. To account for the diffusion-controlled regime, a modification to Kamal equation was implemented [42,43]:

(4) $$\frac{d\alpha }{dt}={\left(k_{1}+k_{2}\alpha^{m}\right)\left(1-\alpha \right)}^{n}\frac{1}{1+exp\left[C\left(\alpha -\alpha_{c}\right)\right]}$$

where C is a constant, and $\alpha_{c}$ is critical conversion. When α reaches $\alpha_{c}$, the reaction becomes diffusion controlled. All fitting parameters were determined using the generalized reduced gradient optimization technique [44].

**Figure 4:**
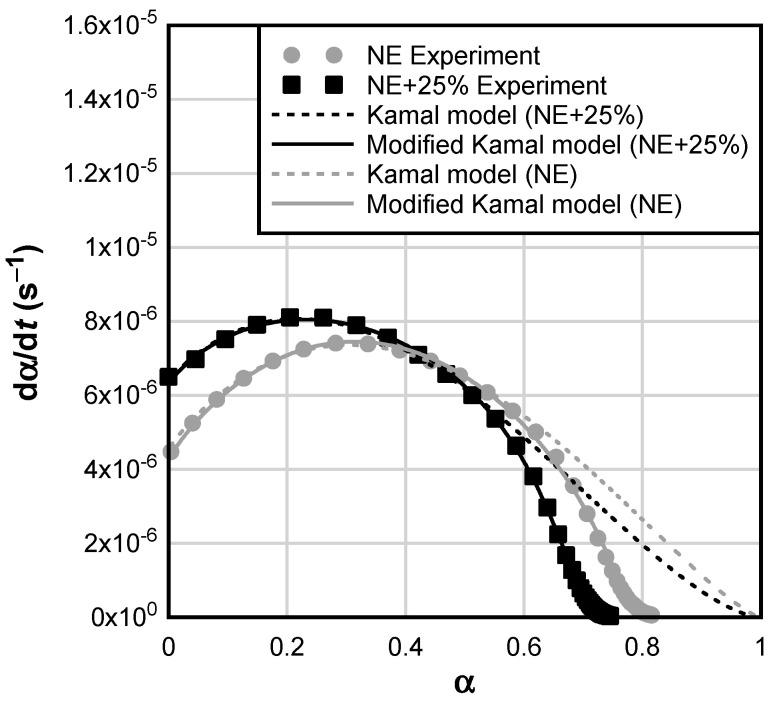
Reaction rate as a function of conversion at 25 °C; the plot shows the comparison between experimental data, Kamal model, and modified Kamal model.

Figure 4 shows the comparison between experimental data, Kamal model, and modified Kamal model fits. It can be observed that Kamal equation can be used to accurately model the initial stages of the curing reaction that are governed by the chemical regime. However, due to the conversion independence of Kamal model, the prediction departs from experimental data at later stages of the reaction. On the contrary, modified Kamal equation takes into consideration the conversion-dependent vitrification effect on the curing reaction, resulting in a better fit. All curing reactions were, therefore, modeled using the modified Kamal equation. Reaction parameters are summarized in Table 4.

**Figure 5:**
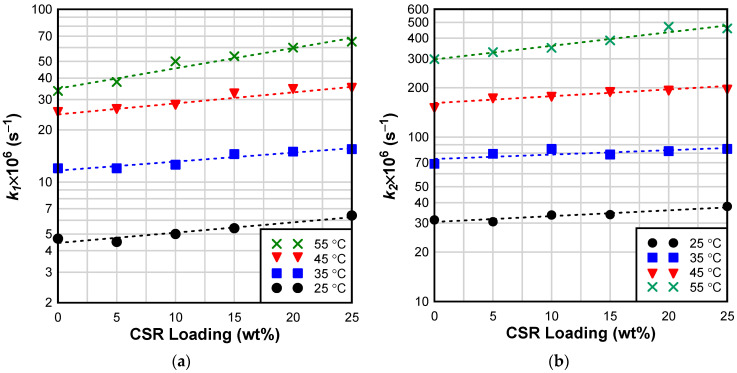
Effect of temperature and CSR loading on (**a**) reaction constant $k_{1}$; (**b**) reaction constant $k_{2}$.

Reaction constants, $k_{1}$ and $k_{2}$, increased with curing temperature and CSR loading confirming the suspected mildly catalytic effect of CSR on the curing reaction (Figure 5). In addition, $\alpha_{c}$ and $\alpha_{max}$ increased with the curing temperature; however, both variables decreased with CSR loading. This is not surprising considering that CSR increases the viscosity of the epoxy, thus more thermal energy is required to maintain the molecular mobility required for continued cure in the diffusion-controlled regime of epoxy/CSR blends than in neat epoxy [45].

Logarithmic form of Arrhenius equation was used to evaluate the temperature dependence of curing reaction for CSR/epoxy blends:

(5) $$\ln\left[k_{i}\left(T\right)\right]=\ln\left[Z_{i}\right]-E_{ai}/RT$$

where $k_{i}\left(T\right)$ is the reaction rate at temperature T; Z is the pre-exponential factor (also known as frequency factor); $E_{a}$ is activation energy; R is gas constant (8.314 J mol^−1^ K^−1^); and e is the natural logarithm base. Terms $E_{a}/R\;$ and Z were determined as slope and intercept of $\ln\left[k_{i}\left(T\right)\right]$ versus 1/T plot. Arrhenius kinetic parameters are summarized in Table 4. The range of activation energies of the tested adhesives is in good agreement with values reported by other researchers for similar epoxy systems [39,46,47,48]. Surprisingly, it appears that activation energy increases with CSR loading, which is inconsistent with the demonstrated catalytic effect of CSR. However, ANOVA analysis of $\ln\left[k_{i}\left(T\right)\right]$ versus 1/T slopes revealed that the apparent differences between the kinetic parameters are not statistically significant. Therefore, although the observed catalytic effect of CSR would indicate a reduction of activation energy, it was not possible to discern a statistically significant effect of CSR on Arrhenius kinetic parameters.

### 4.2. Effect of CSR on Glass Transition Temperature

Glass transition temperature is an important property of ambient-cured adhesives because it establishes their maximum service temperature limit. To determine how CSR affects the $T_{g}$ of epoxy blends, master plots (establishing a theoretical relationship between $T_{g}$ and conversion) were created for NE and epoxy/CSR blends and fitted to the theoretical master curve values using the semi-empirical equation developed by [49]:

(6) $$T_{g}=\frac{\left(1-\alpha \right)T_{g0}+\alpha \left(\Delta C_{p\infty }/\Delta C_{po}\right)T_{g\infty }}{\left(1-\alpha \right)+\left(\Delta C_{p\infty }/\Delta C_{po}\right)\alpha }$$

where α is conversion, $T_{g0}$ and $T_{g\infty }$ are glass transition temperature of the monomer and fully cured samples, respectively. $\Delta C_{p\infty }$ and $\Delta C_{po}$ are the heat capacity changes in J/K (0.55 and 0.85 for NE and NE+25%, respectively).

**Figure 6:**
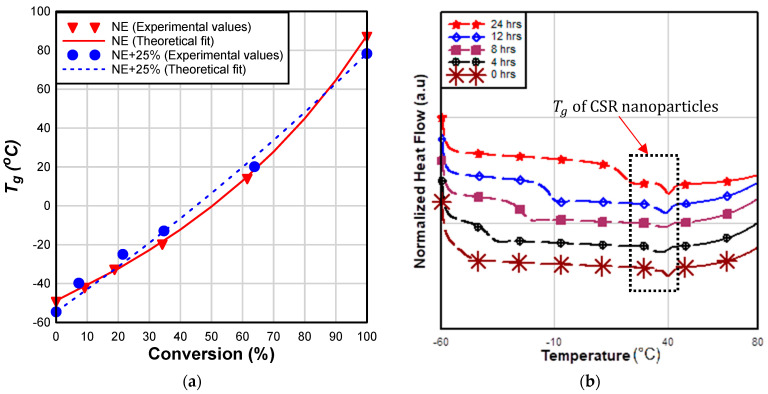
(**a**) Direct comparison of $T_{g}$ between NE and NE+25% groups; (**b**) DSC plot of NE+25% blend (black square shows the $T_{g}$ of CSR nanoparticles).

From Figure 6a, it is clear that there is no significant difference between the master plots of NE and NE+25% groups. A better theoretical fit of master plot values was observed in neat epoxy in comparison to NE+25% group. The $\;T_{g}$ at 0% and 100% conversion of NE+25% samples were observed to be lower than those of neat epoxy; this behavior can be attributed to the higher molecular weight of JER 828 resin compared to that of Epon 826 (Table 2).

NE+25% samples exhibited an endothermic heat-flow peak attributed to the glass transition of CSR nanoparticles (Figure 6b). A similar DSC endothermic peak for CSR nanoparticle samples was reported by Quan and Ivankovic [50]. This transition occurs at a relatively low temperature (≈40 °C), and the $T_{g}$ of the base resin is generally going to be greater than this value. It is not clear what the effect of CSR $T_{g}$ is on the curing kinetics and $T_{g}$ of epoxy/CSR blend.

**Figure 7:**
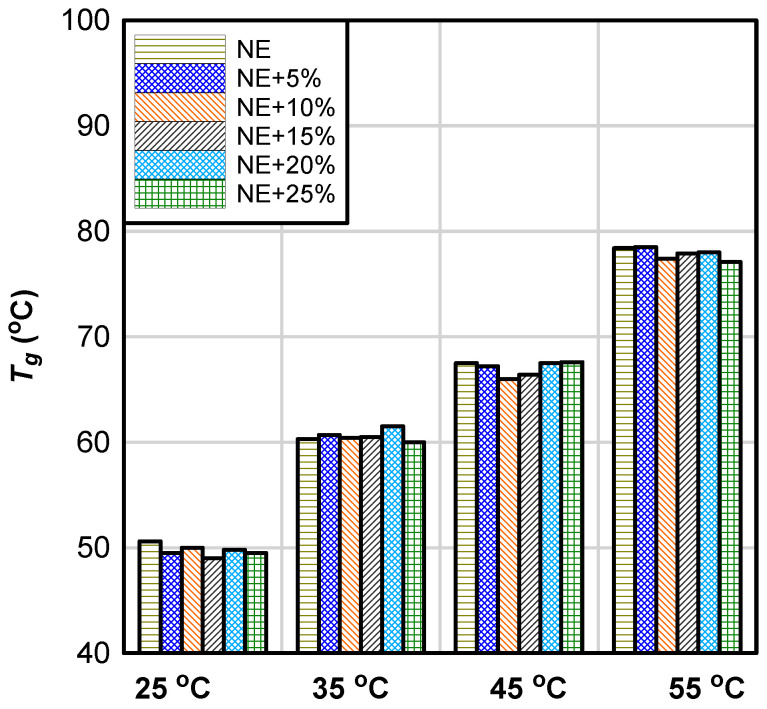
Glass transition temperature of neat and epoxy/CSR blends.

Although CSR has a catalytic effect on the curing reaction, that effect does not extend to $T_{g}$*;* $T_{g}$ remained nearly equal within the tested groups and is primarily dependent on the curing temperature (Figure 7). Therefore, the data indicate that CSR nanoparticles do not affect crosslinking density of the adhesive. The observed negligible effects of CSR on $T_{g}$ in this study is consistent with the findings of other researchers [15,51]. He et al. [52] attributed the unchanged $T_{g}$ of rubber-modified epoxy resin to good phase separation between rubber and resin matrix. The observed negligible effect of CSR on the $T_{g}$ is a major benefit of CSR; other rubber tougheners (as explained in the introduction) reduce the crosslinking density (and, consequently, the $T_{g}$), making these tougheners unsuitable for applications in environments where high $T_{g}$ is required. 

According to the American Concrete Institute design guideline for externally bonded FRP (ACI 440.2R) [53], the maximum permitted service temperature is computed as $T_{g}$ minus 15 °C. The least reported $T_{g}$ (48 °C) for ambient cured samples will allow a maximum service temperature of 33 °C, which is reasonably high for most summer conditions in the United States.

On the other hand, AASHTO [54] guidelines recommend that the glass transition temperature of CFRP systems for concrete bridge elements should be at least 22.2 °C higher than the maximum design temperature for a specific geographic location. Given a reported maximum design temperature ranging between 54.4 °C (Arizona) and 35 °C (Michigan) in the United States, per AASHTO LRFD [55], the minimum recommended $T_{g}$ for CFRP-epoxy system to be used for steel girder bridges with concrete decks ranges between 57.2 °C to 76.6 °C, which implies that ambient curing of epoxy at temperature above 25 °C and 45 °C (Figure 7) is required for the coldest and hottest region of the United States, respectively.

### 4.3. Tensile Properties of Epoxy Adhesives

**Figure 8:**
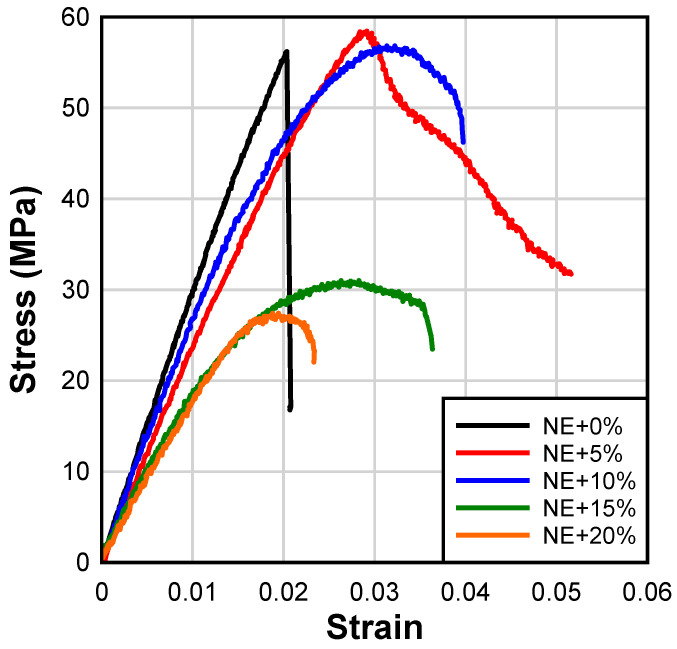
Typical stress–strain curves of adhesives.

Typical stress–strain curves for different epoxy adhesives are shown in Figure 8. As expected, the neat epoxy exhibits linear elastic behavior characterized by a brittle failure. The addition of CSR nanoparticles improves the tensile toughness of the adhesives which is reflected in the increased elongation of CSR-modified epoxies over the neat epoxy. Interestingly, the addition of 5% of CSR results in linear elastic behavior up to the maximum stress, which is followed by a strain-softening behavior. The transition between the linear elastic and plastic part of the stress–strain curve is more gradual at CSR loading ratios exceeding 5%. It is also noted that the addition of CSR starts to significantly affect the tensile strength at loading ratios greater than 10%.

**Figure 9:**
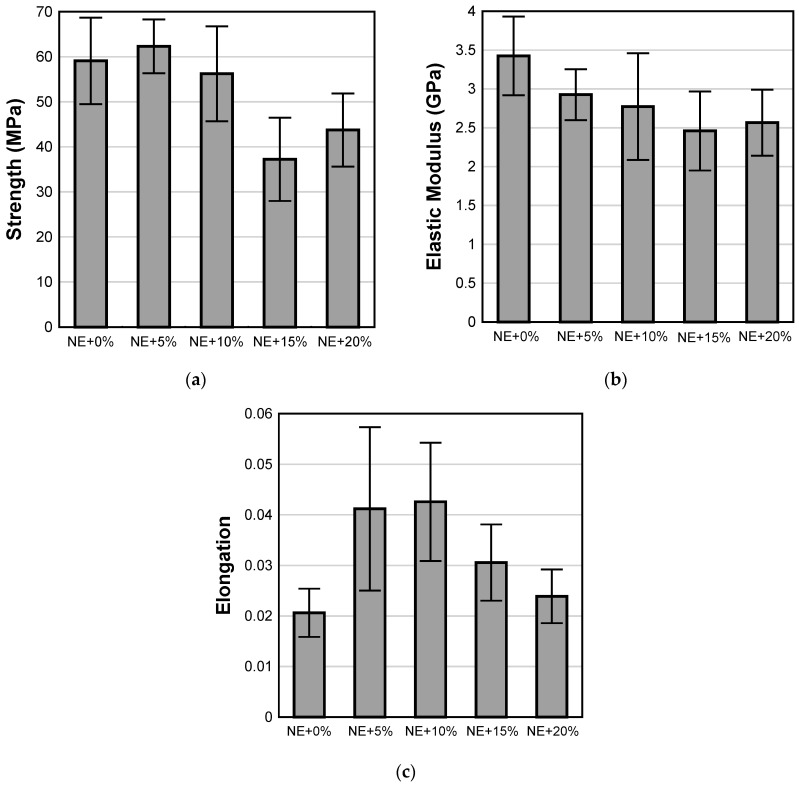
Average values of epoxy adhesives: (**a**) tensile strength; (**b**) elastic modulus; and (**c**) elongation.

Figure 9 shows average values of tensile strength, elastic modulus, and elongation for all epoxy adhesives. The addition of CSR at 5% and 10% loading does not have a statistically significant effect on the tensile strength of the adhesive when compared to the neat epoxy (Figure 9a). However, as discussed previously, a statistically significant loss in strength is observed for loading ratios of 15% and 20%. This suggests that the tensile behavior of the modified epoxy matrix is dominated by CSR which is evident at higher loading ratios [56]. A similar reduction in tensile strength of epoxy resin with an increasing CSR loading ratio was reported by [56,57]. These reductions in tensile strength are attributed to increased stress concentrations due to the high loading of soft inclusions and/or defects originating from the possible agglomeration of CSR nanoparticles at higher loading ratios.

Furthermore, CSR nanoparticles act as compliant inclusions within the brittle neat epoxy matrix which results in a gradual reduction in elastic modulus with an increasing CSR loading ratio (Figure 9b). The addition of CSR resulted in a statistically significant increase in the elongation capacity of epoxy adhesives at failure, particularly at 5% and 10% loading ratios (Figure 9c), which is attributed to the toughening effect of CSR nanoparticles (Figure 1). Increasing the CSR loading ratio over 10%wt. resulted in reductions in elongation although it still remained greater than that observed in the NE+0% group.

### 4.4. Single-Lap Shear Tests

**Figure 10:**
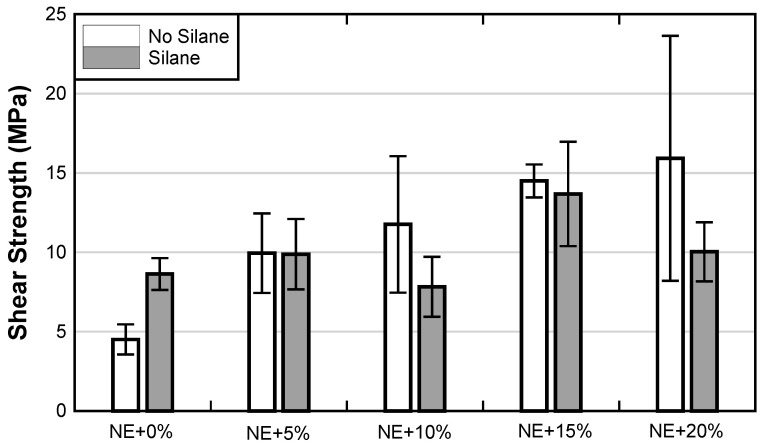
Single-lap shear test results: shear strength was computed by normalizing the maximum recorded load by the area of the adhesive joints.

Average shear strengths of adhesive joints from the single-lap shear experiments are presented in Figure 10. A summary of representative failure modes is included in Table 5. When NE is used without any modifications, lap shear strength is the lowest. Simple treatment of the steel substrate with silane resulted in an approximately 90% increase in the shear strength of the NE+0% adhesive joint. The change in strength was also accompanied by a change in failure mode. NE+0% samples without silane surface treatment failed by separation along the epoxy–steel interface, while the samples with silane-functionalized steel substrate exhibited a mixed failure mode consisting of CFRP-epoxy interfacial separation, cohesive failure of the adhesive, and a small amount of epoxy–steel interfacial failure. The increase in strength with silane functionalization of NE+0% accompanied by a change in failure more clearly indicates that silane surface functionalization is effective in improving the practical adhesion between brittle epoxy and steel substrate.

Addition of 5% CSR to NE results in an approximately 120% improvement in lap shear strength over the NE+0% group without silane treatment. The failure mode of the test specimens in the NE+5% group clearly indicates that damage was distributed within the adhesive layer which is in line with the observed improvement in the adhesive joint strength over the NE+0% group. Silane surface functionalization combined with 5% CSR, however, does not result in further improvement in the adhesive joint strength. Interestingly, the failure mode is quite different between the two groups of NE+5% samples. While a significant portion of the bonded joint without silane failed by interfacial separation along with the epoxy–steel interface, the silane group failed mostly by interfacial separation along with the epoxy–composite interface which indicates that silane surface functionalization was effective at maintaining good adhesion between epoxy and steel.

The NE+10% group did not exhibit statistically significant improvement in the joint strength over the NE+5% group. In fact, the silane-treated samples in the NE+10% group had a reduction in strength in comparison to their NE+5% counterpart, which was also followed by a drastic shift in failure mode—from the epoxy–composite interface in the NE+5% group, to epoxy–steel interface in NE+10% group. It is not clear why this shift in failure mode occurred.

Further improvements in joint strength were observed with the addition of 15 and 20% of CSR. Even though the addition of 15% and 20% of CSR to the NE resulted in reduced strength (Figure 10), these resins yield an improvement of approximately 250% in lap shear bond strength over NE+0% (without silane). However, it is not clear how reliable the bond strength improvement is in the NE+20% group without silane surface treatment due to the large variation in test data. Silane functionalization for both 15% and 20% CSR loading ratios did not prove to be beneficial. In fact, the NE+20% group with silane surface treatment exhibited a reduction in joint strength over its “non-silane” counterpart. Interestingly, the epoxy–steel interfacial separation remained characteristic for the silane functionalized joints. In general, failure modes in NE+15% and NE+20% remained relatively similar to those observed in the NE+10% group. At CSR loading ratios above 10%, the amount of entrapped air bubbles/voids along the bondline was observed to increase, hence, single-lap shear test was terminated at NE+20% due to workability issues.

## 5. Summary and Conclusions

Ambient-cured epoxy and wet-layup CFRP used for concrete strengthening are not necessarily appropriate for repair and strengthening of steel structures. As a potential means of addressing the backlog of 600,000 steel bridges in need of repair in the United States alone, this study explored altering existing CFRP strengthening technology with CSR nanoparticles to accommodate applications in steel structures. The objective of the study was to evaluate the effect of CSR nanoparticles on curing kinetics, glass transition temperature, and mechanical properties of epoxy. In addition, the study investigated the effects of CSR nanoparticles and silane surface treatment on CFRP/steel adhesive joint strength. The following conclusions can be drawn from the presented results:

- CSR nanoparticles have a mild catalytic effect on the curing reaction of epoxy, but no notable effect on Arrhenius kinetic parameters or $T_{g}$ was observed. The addition of CSR decreased the critical and maximum conversion of epoxy/CSR blend, likely due to the effects of increased viscosity on the diffusion-controlled curing kinetics of the epoxy/CSR blend. Owing to the significance of diffusion-controlled regime in ambient-cured epoxy adhesives, a modified Kamal equation was found to be more appropriate for modeling the initial (i.e., chemical) and final (i.e., diffusion controlled) stages of the curing reaction for both NE and epoxy/CSR blends.
- The addition of CSR nanoparticles to epoxy resin increased the elongation capacity of the adhesive by up to 125%. This effect was most significantly pronounced at 5%wt. and 10%wt. CSR loading ratios.
- The addition of CSR nanoparticles reduced strength and elastic modulus by up to 28% and 24%, respectively, when compared to the base resin. This effect became significant at the loading ratios of 15% and 20%.
- Modification of NE with CSR nanoparticles increased the single-lap shear strength of CFRP-steel joints by 117 to 269%. Maximum joint strengths were observed at CSR loading ratios of 15% and 20%; this represents a 250% increase in joint strength over the NE adhesive.
- Silane surface treatment is effective in improving the lap shear strength of CFRP/steel joints made with NE. At a higher CSR loading ratio, silane surface treatment had detrimental effects on the shear strength of CFRP/steel joints and did not offer significant benefits over NE+0% group joints.

## 6. Practical Recommendations

Based on the results of this study, 5 to 20%wt. CSR loading ratio is efficient in improving the ductility of epoxy and can be used to modify the existing wet-layup CFRP systems for structural steel applications. Incorporating 5 to 10%wt. CSR loading is the most effective in increasing the elongation of epoxy, with no effects on the tensile strength. Higher CSR loading ratios are characterized by reduced tensile strength, elongation, and elastic modulus. For optimal shear strength of steel/CFRP joints, CSR loading ratio of 20%wt. is recommended without using silane to treat the steel surface. The use of silane for structural steel surfaces is not recommended for epoxy/CSR blend above 5% CSR because it decreases the shear strength of steel/CFRP joints. Since all CSR loading used in this study has no significant effect on the curing kinetics and glass transition of epoxy, no significant cost can be attributed to time due to the use of CSR. The cost effectiveness of adopting this technology for strengthening (and retrofitting) steel bridges will largely depend on the degree of shear strengthening required, the cost of CSR nanoparticles, the comparative cost of CFRP sheets (as opposed to laminates), and the comparative cost of competing technologies (e.g., the use of steel plates).

To increase confidence in the proposed technology, future work is required to understand the long-term performance of CSR-modified epoxy joints after exposure to environments typically encountered by infrastructure, particularly freeze–thaw, moisture, high temperature, and combinations thereof. The effect of silane might be more significant when considering the durability of steel/CFRP joints, especially when considering exposure to environmental stressors. The effect of sustained and fatigue loading on CSR-modified epoxy joints is also deemed a critical issue. Finally, additional research should be conducted to understand the interplay between CSR nanoparticles, silane surface treatment and the effect of steel substrate surface preparation (e.g., grit blasting, etching, grinding, etc.).

## Figures and Tables

**Table 1:** Low-carbon steel properties.

Property	Value
Yield strength	370 MPa
Elongation	23%
Fabrication	Cold worked
Temper rating	Hardened
Hardness	Rockwell B70
Material composition	Iron 98.06–99.42%
	Carbon 0.13–0.20%
	Manganese 0.30–0.90%
	Phosphorus 0.04% Max.
	Silicon 0.15–0.30%
	Sulfur 0.50% Max.

**Table 2:** Properties of epoxy adhesive components.

Product	Viscosity (cps)	Epoxide Equivalent Weight (EEW) (g/eq)	Amine Hydrogen Equivalent Weight (AHEW) (g/eq)
Epon 826	450 @ 50 °C	178–186	n/a
Jeffamine D-230	9.5 @ 25 °C *	n/a	60
CSR Masterbatch	3000 @ 50 °C	243	n/a
JER828 **	12,000 to 15,000 @ 25 °C	184–194	n/a

* Kinematic viscosity in cSt. ** base resin in CSR Masterbatch. (n/a: Jeffamine doesn’t have epoxide groups. Likewise, Epon, CSR masterbatch and JER828 do not have amine groups.)

**Table 3:** Dry fiber and CFRP properties.

Property	Dry Fiber	CFRP
	Test Value	
Tensile strength	4.0 GPa	985 MPa
Tensile modulus	250 GPa	95 GPa
Elongation at rupture	1.7%	1.0%
Density	1.74 g/cm^3^	n/a
Weight/unit area	644 g/m^2^	n/a
Nominal thickness		1.0 mm

**Table 4:** Isothermal curing kinetic parameters.

Adhesive	$T_{cure}$ (°C)	$\alpha_{max}$	$\alpha_{c}$	C	$k_{1}\;\times$ 10^6^ (s^−1^)	$k_{2}\;\times$ 10^6^ (s^−1^)	m	n	m+n	$E_{a1}$ (kJ/mol)	$Z_{1}\;\times$ 10^5^ (GW/g)	$E_{a2}$ (kJ/mol)	$Z_{2}\;\times$ 10^4^ (GW/g)
NE	25	0.82	0.74	39.7	4.7	31.4	1.09	1.59	2.68	54.5	1.84	61.5	18.6
	35	0.91	0.85	52.8	12.0	68.9	1.04	1.43	2.47				
	45	0.93	0.88	61.0	25.0	153.5	1.00	1.60	2.60				
	55	0.96	0.93	90.6	33.7	298.3	0.72	1.60	2.32				
NE+5%	25	0.80	0.73	40.8	4.5	30.6	1.07	1.60	2.67	58.4	8.68	64.6	67.0
	35	0.87	0.80	47.3	12.0	79.2	1.14	1.67	2.82				
	45	0.90	0.85	64.2	26.0	175.6	1.12	1.80	2.92				
	55	0.93	0.89	81.0	38.0	329.1	0.80	1.80	2.60				
NE+10%	25	0.81	0.72	43.5	5.0	33.6	1.11	1.70	2.81	62.5	47.6	63.3	43.5
	35	0.84	0.77	46.8	12.6	84.9	1.14	1.86	3.00				
	45	0.89	0.84	57.7	27.5	179.6	1.09	1.84	2.93				
	55	0.93	0.89	78.7	50.0	349.4	0.84	1.86	2.70				
NE+15%	25	0.78	0.68	41.6	5.4	33.8	1.09	1.80	2.89	62.6	54.5	66.8	173.3
	35	0.84	0.77	48.3	14.5	78.5	1.07	1.76	2.83				
	45	0.87	0.82	59.8	32.0	191.8	1.09	1.94	3.04				
	55	0.93	0.89	77.9	53.5	388.0	0.99	1.86	2.85				
NE+20%	25 *	n/a	n/a	n/a	n/a	n/a	n/a	n/a	n/a	n/a	n/a	n/a	n/a
	35	0.86	0.79	48.9	15.0	82.3	1.15	1.73	2.87				
	45	0.89	0.83	58.1	34.0	195.6	1.15	1.94	3.09				
	55	0.94	0.90	81.3	60.0	470.4	1.14	1.91	3.05				
NE+25%	25	0.75	0.67	44.5	6.4	37.9	1.12	1.97	3.09	63.2	77.1	67.7	269.4
	35	0.80	0.73	48.0	15.5	84.4	1.08	1.96	3.04				
	45	0.86	0.81	58.6	34.5	198.3	1.09	1.99	3.08				
	55	0.88	0.83	74.9	65.0	458.6	0.91	2.30	3.20				

* The test was terminated prematurely (before reaching asymptotic heat flow); therefore, the fitting constants could not be reliably determined.

**Table 5:** Summary of representative failure modes.

Test Group	No Silane		Silane	
	CFRP Side	Steel Side	CFRP Side	Steel Side
NE+0%	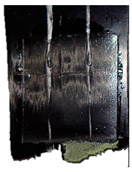	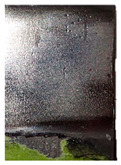	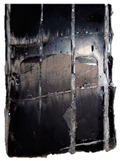	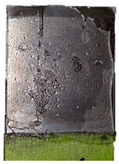
NE+5%	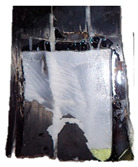	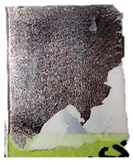	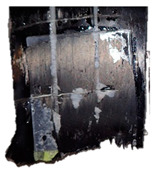	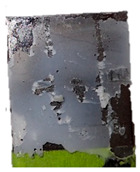
NE+10%	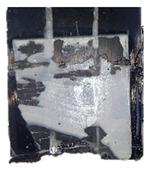	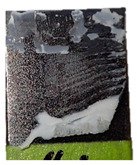	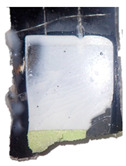	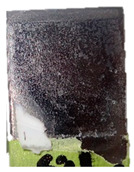
NE+15%	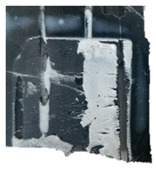	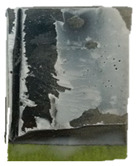	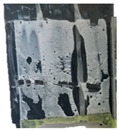	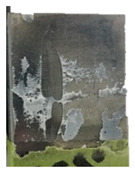
NE+20%	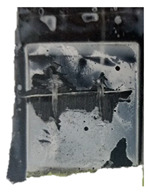	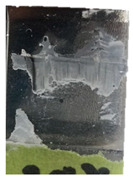	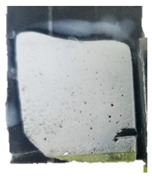	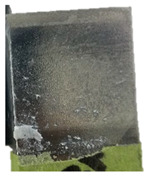

## Data Availability

Data, models, or code that support the findings of this study are available from the corresponding author upon reasonable request.
